# New Appliances and Things Medical

**Published:** 1900-11-03

**Authors:** 


### NEW APPLIANCES AND THINGS MEDICAL.

[We shall be glad to receive, at our Office, 23 & 29 Southampton Street, Strand, London, W.C., from the manufacturers, specimens of all new preparations and appliances which may be brought out from time to time.]

### BLANCHE LEIGH SOAP.

(London Agency, 126 Oxford Street, W.)

We have examined several soaps belonging to the series now being introduced by Madame Blanche Leigh. The three soaps to which we would draw attention are—(1) The Transvaal Soap; (2) Marie Antoinette; (3) Beautylene; (4) Children's Soap. The Blanche Leigh soaps and toilette preparations are well known on the Continent, where they are highly appreciated, partly because they are pure, but chiefly because their inventor has grasped the importance of utilising scientific methods and the therapeutic properties of medicaments of known value. For instance, Ichthyol, a most efficacious dermatological application, has for years past been, perhaps, the most appreciated therapeutic agent in the skin specialist's armamentarium. It has been combined by them in every possible form of ointment, lotion, cream, and paint, but the result has seldom been successful from an artistic point of view. The combination of efficacy with artistic pharmacy has been left for Madame Leigh. Her soaps, which contain this valuable medicament, are no less efficacious because the naturally unpleasant odour of Ichthyol is replaced by the agreeable odour of the choicest flowers. Ichthyol is a sulphur-containing body, and has undoubted influence on the healthy growth of skin; and being in a certain sense antiseptic it removes or prevents such blemishes as comedones, pimples, and unbecoming rashes. Whether for nursery or general use the Blanche Leigh Soaps are strongly to be recommended. They form a beautiful lather, are free from excess of alkali, and exercise a healthy cleansing effect upon the integuments.

### STRENBO.

(Liebig's Extract of Meat) Premier Chef Brand, Strenbo Co., 64 Corporation Street, Manchester.

Strenbo is a concentrated extract of meat prepared by a modified process which embraces the chief advantages of Liebig's method, combined with those derived from recent investigations with regard to the preservation of the nutritive elements of meat in a soluble form. As is well known, the original extracts of meat made on Liebig's principle contained the soluble extractives, but were comparatively free from the principles of meat which can be directly regarded as foods. Strenbo to a degree reached by few other preparations of this nature—is a real food and a powerful stimulant.

### BRANDY AND WHISKEY.

(Innes, Smith and Co., 83 High Street, Birmingham.)

We have received samples of the above spirits from Messrs. Innes, Smith and Co. The brandy, which is a fine old cognac, appears a spirit of first-rate quality, well matured, and free from the crude alcohols of the higher series which destroy the value of cheap spirits of all sorts as therapeutic media. We highly recommend it as a reliable brandy for invalid use. The whiskey, which is a very pronounced Scotch spirit, with strong flavouring of peat smoke, will be much appreciated by those who like this breezy reminder of the Highlands. The spirit itself is undoubtedly a well-matured variety, and, like the cognac, may safely be ordered by medical men for their patients.

